# Impact of re-definition of paroxysmal and persistent atrial fibrillation in the 2012 and 2016 European Society of Cardiology atrial fibrillation guidelines on outcomes after pulmonary vein isolation

**DOI:** 10.1007/s10840-020-00710-4

**Published:** 2020-03-02

**Authors:** Karolina Weinmann, Deniz Aktolga, Alexander Pott, Carlo Bothner, Manuel Rattka, Tilman Stephan, Wolfgang Rottbauer, Tillman Dahme

**Affiliations:** Department of Medicine II, Ulm University Medical Center (Universitätsklinikum Ulm), Albert-Einstein-Allee 23, Ulm, Germany

**Keywords:** Atrial fibrillation, Paroxysmal, Persistent, Guidelines, Ablation, Outcome

## Abstract

**Purpose:**

In the 2016 European Society of Cardiology (ESC) guidelines for the management of atrial fibrillation (AF), the definition of AF type has been modified compared with the 2010 guidelines and its 2012 focused update. We compared the difference of single procedure outcomes using the definitions before and after 2016 on a cohort of patients with AF undergoing AF ablation.

**Methods:**

Consecutive AF ablation patients with paroxysmal or persistent AF were retrospectively reclassified applying the 2010, 2012, and 2016 ESC definitions on AF type.

**Results:**

We included a total of 628 patients. Applying the 2010 ESC AF guidelines definition, 68% of patients were paroxysmal while according to the 2016 ESC AF guidelines, the proportion increased to 87%. Applying the 2010 ESC guidelines definition, recurrence rates of paroxysmal and persistent AF patients differ significantly (log-rank *p* < 0.001). Applying the 2012 focused update and the 2016 ESC AF guidelines, recurrence rates do not differ significantly. In a cox regression model applying the 2010 guidelines, persistent AF is the only independent predictor of AF recurrence in our cohort. However, when applying the 2016 guidelines, persistent AF is no longer a predictor of AF recurrence.

**Conclusions:**

The revised definition of AF types in the 2016 ESC AF guidelines leads to a marked shift from persistent to paroxysmal AF. It appears that the old definition provided a better separator to predict rhythm outcome after AF ablation.

**Electronic supplementary material:**

The online version of this article (10.1007/s10840-020-00710-4) contains supplementary material, which is available to authorized users.

## Introduction

Guidelines summarize and evaluate current knowledge on a particular topic to determine evidence-based standards and serve as guidance for physicians for therapeutic management. In the 2016 European Society of Cardiology (ESC) guidelines for the management of atrial fibrillation (AF), the definition of AF type has been modified compared with the 2010 guidelines for the management of AF and its 2012 focused update [1–3]. While the definition of first diagnosed, long-standing persistent and permanent AF remained unchanged from 2010 to date, the definition of paroxysmal and persistent AF has been modified twice. AF in patients with spontaneous conversion into sinus rhythm within 7 days is defined as paroxysmal, whereas patients without spontaneous conversion into sinus rhythm within 7 days or need for cardioversion are labeled persistent in the 2010 ESC guidelines [2]. In the 2012 focused update, patients with active conversion into sinus rhythm either with drugs or by direct current cardioversion within 48 h that would have been persistent according to the 2010 guidelines were labeled paroxysmal [2]. In the 2016 ESC guidelines, all patients with AF lasting up to 7 days and spontaneous conversion or termination by cardioversion either with drugs or by direct current cardioversion are labeled paroxysmal. Only AF episodes lasting longer than 7 days are now defined as persistent [3]. Patients with active conversion to sinus rhythm within 7 days after onset of AF are labeled paroxysmal in the 2016 ESC guidelines because their probability of spontaneous conversion, had they not been cardioverted, is deemed high. That means that the new guidelines aim to adjust for the probability that patients labeled as paroxysmal would have a higher chance of spontaneous conversion without intervention. Thus, the difference between paroxysmal patients with spontaneous conversion within 7 days and patients with very early active conversion is likely rather the severity of symptoms than underlying pathophysiological differences.

During its natural course, AF usually progresses from paroxysmal to persistent and finally to permanent AF [4]. However, it is well known that in some patients, AF primarily occurs as persistent AF, while other patients remain in paroxysmal AF for many years or even decades [5]. Nevertheless, many studies distinguish these two entities to evaluate outcome and conclude diagnostic, therapeutic, and prognostic implications [6–8]. Pulmonary vein isolation (PVI) has become an established therapy to treat symptomatic, paroxysmal, and also persistent AF [9–11]. Recurrence rates after PVI differ reproducibly between patients with paroxysmal and persistent AF [6, 7, 12]. However, whether the modification of the definition of paroxysmal and persistent AF has an impact on the outcome of AF ablation has not been investigated yet. We compared the outcome of single-procedure PVI with the cryoballoon applying the definition of the 2010 and 2016 ESC guidelines as well as the 2012 focused update.

## Methods

### Study population

We included consecutive patients with paroxysmal or persistent AF, who underwent cryoballoon PVI as a first procedure from January 2013 to November 2018 at Ulm University Medical Center. Patients were retrospectively reclassified as paroxysmal or persistent according to the 2010 ESC guideline, 2012 focused update, and 2016 ESC guideline definition of AF type. Written informed consent was obtained from each patient prior to the procedure and the protocol was approved by our local Ethics Committee. The investigation conforms with the principles outlined in the Declaration of Helsinki. The exclusion criteria were long-standing persistent AF, previous left atrial (LA) ablation, LA diameter > 55 mm, uncontrolled heart failure (NYHA class IV), and severe valvular disease. The first 224 patients were treated with a fixed freeze protocol and the last 395 patients were treated with a time to isolation guided freeze protocol [13, 14].

### Pre-procedural management

Pre-procedural intracardial thrombi were ruled out by transesophageal echocardiography (TEE). Oral anticoagulation with vitamin K antagonists was not interrupted and a target INR was aimed at 2.0–3.0. Novel oral anticoagulants (NOAKs) were discontinued 24 h prior to the procedure.

### Ablation procedure

All procedures were performed in deep sedation using midazolam and nurse-assisted propofol administration. Analgesia was achieved by fentanyl bolus administration. A 6F steerable decapolar catheter was placed in the coronary sinus (CS). LA access was obtained by a single transseptal puncture under fluoroscopy guidance using a modified Brockenbrough catheter and a 2H transseptal needle (Maslanka, Tuttlingen, Germany). Thereafter, a heparin bolus was administrated, targeting an activated clotting time of > 300 s. A guidewire was advanced in the left superior pulmonary vein (PV) and a 12F steerable sheath (Flexcath advance, Medtronic, USA) was positioned in the left LA. All PV ostia were visualized by PV angiography. Then a 28-mm cryoballoon (Arctic Front Advance, Medtronic, USA) was introduced in the LA and guided to the target PV over a 20-mm spiral mapping catheter (Achieve, Medtronic). Complete occlusion of the respective PV by the inflated cryoballoon was verified by selective dye injection. No backflow to the atrium was considered as optimal occlusion of the PV. The esophageal temperature was monitored by a temperature probe (Sensitherm; St. Jude Medical Inc., St Paul, MN, USA or S-Cath; Circa Scientific Inc., USA) that was nasally placed in the esophagus, at the closest possible proximity to the ablation site. A luminal esophageal temperature of 15–20 °C was the cut-off temperature, leading to abortion of the freeze cycle. Phrenic nerve function was monitored by phrenic nerve stimulation and detection of compound motor action potentials (CMAP), as well palpation of diaphragm contractions during ablation of the right sided PVs [13, 14].

### Post-procedural management

Pericardial effusion was excluded by echocardiography following the procedure and before hospital discharge. NOAKs were continued at the evening of the procedure day. Anticoagulation was continued for at least 2 months or longer depending on the individual CHA_2_DS_2_-VASc score. Patients were continuously monitored including electrocardiogram (ECG) for 24 h. A 12-lead surface ECG and a 24-h Holter-ECG was performed before discharge.

### Follow-up

Patients were scheduled in our outpatient clinic for follow-up visits at 1, 3, 6, 12, 18, and 24 months after the procedure including 12-lead surface ECG and 7-day Holter monitoring. Any documented episode of AF or atrial tachyarrhythmia longer than 30 s was considered as recurrence. Patients with suspected recurrence because of specific symptoms were monitored more frequently.

### Statistical analysis

Statistical analyses were performed using SPSS® software (SPSS, V25, Chicago, IL, USA). Categorical variables are described as absolute and relative frequencies and continuous variables are expressed as mean ± SD. Event-free survival was estimated using Kaplan-Meier evaluation and statistically compared by log-rank test. Cox proportional hazards models were applied to assess the effect of potential risk factors of AF recurrence. A *p* value < 0.05 was considered statistically significant.

## Results

### Type of AF

We included 628 patients who underwent CB-PVI as the index procedure for symptomatic AF. Mean age was 66.2 ± 10.8 years and 43% of the patients were female. Applying the 2010 ESC AF guidelines definition, 68% (425/628) of patients were categorized as paroxysmal and 32% (203/628) as persistent AF. According to the 2012 focused update, 77% (485/628) are labeled paroxysmal and 23% (143/628) are persistent AF. Applying the 2016 ESC AF guidelines, the proportion of patients with paroxysmal AF increased to 87% (546/628) of patients and the patients with persistent AF decreased to 13% (82/628). Baseline characteristics of the total cohort and the comparison of the baseline characteristics regarding paroxysmal and persistent AF according to the 2010 and 2016 definition are listed in Table 1, baseline characteristics according to the 2012 focused update are shown in supplementary Table 1. Age, left ventricular ejection fraction (LVEF), LA diameter, and CHA_2_DS_2_-VASc score differ significantly between paroxysmal and persistent AF patients according to the 2010, 2012, and 2016 AF type classification.

**Table 1:** Comparison of baseline characteristics of patients with paroxysmal and persistent AF classified after the 2010 and 2016 ESC guidelines definition

	Total	2010 ESC guidelines		*p*	2016 ESC guidelines		*p*
	(*n* = 628)	Paroxysmal (*n* = 425)	Persistent (*n* = 203)		Paroxysmal (*n* = 546)	Persistent (*n* = 82)	
Female gender	272 (43)	191 (45)	80 (39)	0.2	228 (42)	43 (52)	0.1
Age (years)	66.2 ± 10.8	65.4 ± 11.4	67.9 ± 9.3	*0.007*	65.7 ± 10.9	69.4 ± 9.3	*0.005*
Hypertension	457 (79)	298 (70)	159 (78)	0.08	390 (71)	67 (82)	0.1
Dyslipidemia	330 (57.2)	217 (51)	113 (56)	0.4	285 (52)	45 (55)	0.8
Diabetes mellitus	103 (18)	64 (15)	39 (19)	0.2	84 (15)	19 (23)	0.1
Coronary artery disease	206 (36)	129 (30)	77 (38)	0.1	179 (33)	27 (33)	0.8
Peripheral vascular disease	176 (30)	120 (28)	56 (28)	0.7	151 (28)	25 (30)	0.7
Oral Anticoagulation	521 (90)	339 (80)	182 (90)	*0.003*	446 (82)	75 (91)	*0.04*
Other drugs							
Beta blockers	490 (85)	322 (76)	171 (84)	*0.02*	418 (77)	72 (88)	*0.03*
Calcium antagonists	133 (23)	92 (22)	41 (20)	0.8	116 (21)	17 (21)	0.9
LVEF (%)	58.7 ± 15.7	62.3 ± 14.2	51.9 ± 16.2	*< 0.001*	59.5 ± 15.7	54.3 ± 15.4	*0.046*
Left atrial diameter (mm)	43.2 ± 11.4	41.5 ± 11.9	46.5 ± 9.7	*< 0.001*	42.8 ± 11.6	45.7 ± 10.3	*0.02*
CHA_2_DS_2_-VASc score	2.8 ± 1.6	2.7 ± 1.6	3.2 ± 1.6	*< 0.001*	2.7 ± 1.6	3.4 ± 1.5	*< 0.001*
BMI (kg/m^2^)	28.6 ± 15.7	28.5 ± 5.3	28.9 ± 5.5	0.4	28.5 ± 5.3	29.9 ± 5.8	0.5

Categorical variables are expressed as absolute and percentage (in parentheses). Continuous variables are expressed as mean ± SD. *AF*, atrial fibrillation; *BMI*, body mass index; *LVEF*, left ventricular ejection fraction. Significant *p*-values (< 0.05) indicated in italics

Applying the 2010 and 2016 definitions of AF type to our cohort, 123 patients shift from the 2010 persistent group to the 2016 paroxysmal group while 424 patients remain in the paroxysmal group and 81 patients are persistent according to both guidelines. To characterize the patients that switch from persistent to paroxysmal and those that maintain their paroxysmal or persistent state, we compared baseline characteristics of all patients. The cohort that switches from persistent to paroxysmal has a higher fraction of male patients. Patients from paroxysmal, over classification switching and persistent AF patients show an increasing amount of cardiovascular risk factors. In contrast, coronary artery disease is most common in classification switching patients. Patients that maintain their persistent AF state show the highest CHA_2_DS_2_-VASc score and consequently the highest prescription of oral anticoagulation. Remarkably, classification switching patients show worst LVEF and largest LA diameter (Table 2).

**Table 2:** Patients that change from persistent to paroxysmal in comparison to patients without changing groups applying 2016 ESC guidelines

	Group 1	Group 2	Group 3	*p* value		
ESC 2010 guidelines	Paroxysmal	Persistent	Persistent			
ESC 2016 guidelines	Paroxysmal	Paroxysmal	Persistent	1 vs. 2	2 vs. 3	1 vs. 3
Patients	424	123	81			
Female gender	191 (45)	37 (30)	43 (53)	*0.003*	*0.001*	0.2
Age (years)	65.4 ± 11.4	66.9 ± 9.2	69.4 ± 9.3	0.1	0.07	*0.003*
Hypertension	298 (70)	92 (75)	67 (83)	0.3	0.4	0.08
Dyslipidemia	217 (51)	68 (55)	45 (56)	0.4	0.8	0.7
Diabetes mellitus	64 (15)	20 (16)	19 (23)	0.8	0.3	0.1
Coronary artery disease	129 (30)	50 (41)	27 (33)	*0.03*	0.2	0.8
Peripheral vascular disease	120 (28)	31 (25)	25 (31)	0.5	0.5	0.8
Oral anticoagulation	339 (80)	107 (87)	75 (93)	0.1	0.3	*0.01*
Other drugs						
Beta blockers	319 (75)	99 (80)	72 (89)	0.3	0.2	0.06
Calcium antagonists	92 (22)	24 (20)	17 (21)	0.7	0.9	0.9
LVEF (%)	62.3 ± 14.2	50.0 ± 16.7	54.3 ± 15.4	*< 0.0001*	0.1	*< 0.0001*
Left atrial diameter (mm)	41.5 ± 11.9	47.0 ± 9.2	45.7 ± 10.3	*< 0.0001*	0.4	*0.007*
CHA_2_DS_2_-VASc score	2.7 ± 1.6	*3.0* ± 1.6	3.4 ± 1.5	0.07	0.07	*< 0.0001*
BMI (kg/m^2^)	28.5 ± 5.3	28.8 ± 5.3	30.0 ± 5.8	0.5	0.9	0.4

Categorical variables are expressed as absolute and percentage (in parentheses). Continuous variables are expressed as mean ± SD. *AF*, atrial fibrillation; *BMI*, body mass index; *LVEF*, left ventricular ejection fraction. Significant *p*-values (< 0.05) indicated in italics

### Procedural characteristics

All patients were treated with a PVI only strategy with the cryoballoon, without any additional ablation targets. In the 628 patients, we identified 2476 pulmonary veins; all of these were isolated with the cryoballoon without any additional touch-up ablations. Comparison of procedural data of paroxysmal and persistent patients according to the 2010 and 2016 definition is shown in Table 3.

**Table 3:** Procedural characteristics

	Total	2010 ESC guidelines		*p*	2016 ESC guidelines		*p*
		Paroxysmal	Persistent		Paroxysmal	Persistent	
Total procedural time (min)	98.5 ± 35.8	96.2 ± 36.5	102.8 ± 33.6	*0.03*	97.5 ± 35.8	103.4 ± 34.5	0.2
Mean number of freeze—thaw cycles in							
LSPV	1.9 ± 1.0	1.9 ± 0.9	1.9 ± 1.0	0.6	1.9 ± 1.0	1.95 ± 1.1	0.5
LIPV	1.6 ± 0.8	1.6 ± 0.7	1.7 ± 0.9	0.1	1.6 ± 0.8	1.6 ± 0.7	0.8
RSPV	1.7 ± 0.7	1.6 ± 0.7	1.8 ± 0.8	*0.005*	1.7 ± 0.7	1.8 ± 0.8	0.1
RIPV	1.9 ± 0.9	1.9 ± 1.0	1.8 ± 0.8	0.2	1.9 ± 1.0	1.9 ± 0.8	0.9
Mean minimal temperature (°C) in							
LSPV	− 49.5 ± 31.4	− 50.1 ± 38.2	− 48.4 ± 5.4	0.5	− 49.7 ± 33.6	− 48.6 ± 5.3	0.8
LIPV	− 45.4 ± 31.5	− 46.0 ± 37.9	− 44.1 ± 8.8	0.5	− 45.6 ± 33.5	− 44.0 ± 12.0	0.7
RSPV	− 49.7 ± 7.1	− 49.9 ± 6.1	− 49.2 ± 8.9	0.3	− 49.7 ± 7.3	− 49.4 ± 6.3	0.7
RIPV	− 47.6 ± 8.2	− 47.6 ± 8.9	− 47.7 ± 6.6	0.9	− 47.7 ± 8.4	− 47.2 ± 6.4	0.7

Values are expressed as mean ± SD. *LSPV*, left superior pulmonary vein; *LIPV*, left inferior pulmonary vein; *RSPV*, right superior pulmonary vein; *RIPV*, right inferior pulmonary vein. Significant *p*-values (< 0.05) indicated in italics

### Follow-up and AF recurrence

In total, 618 patients attended clinical follow-up with a mean duration of 18.0 ± 15.3 months, only ten patients (0.5%) were lost to follow-up.

**Fig. 1:**
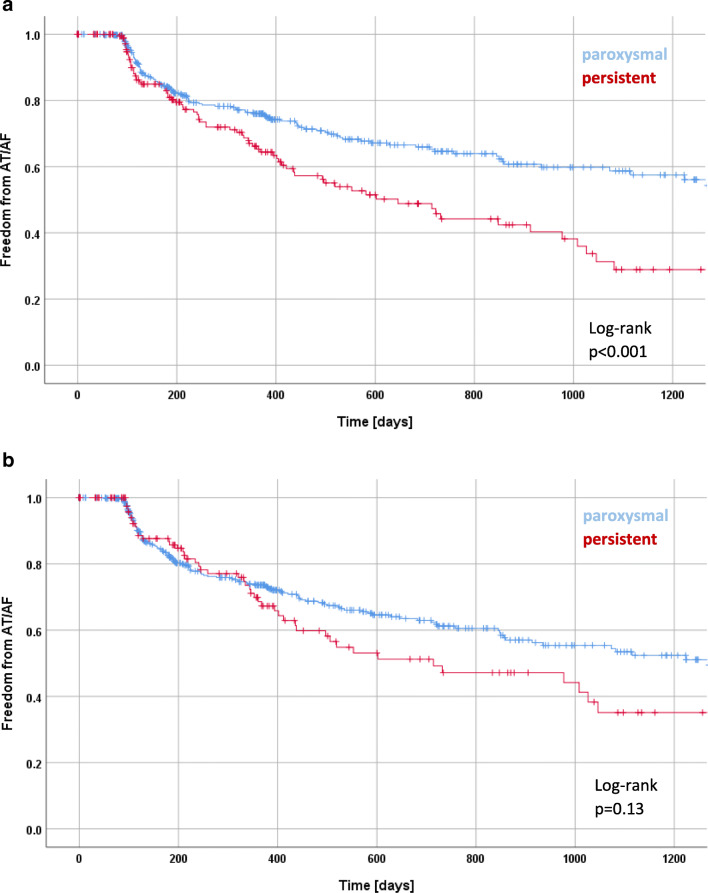

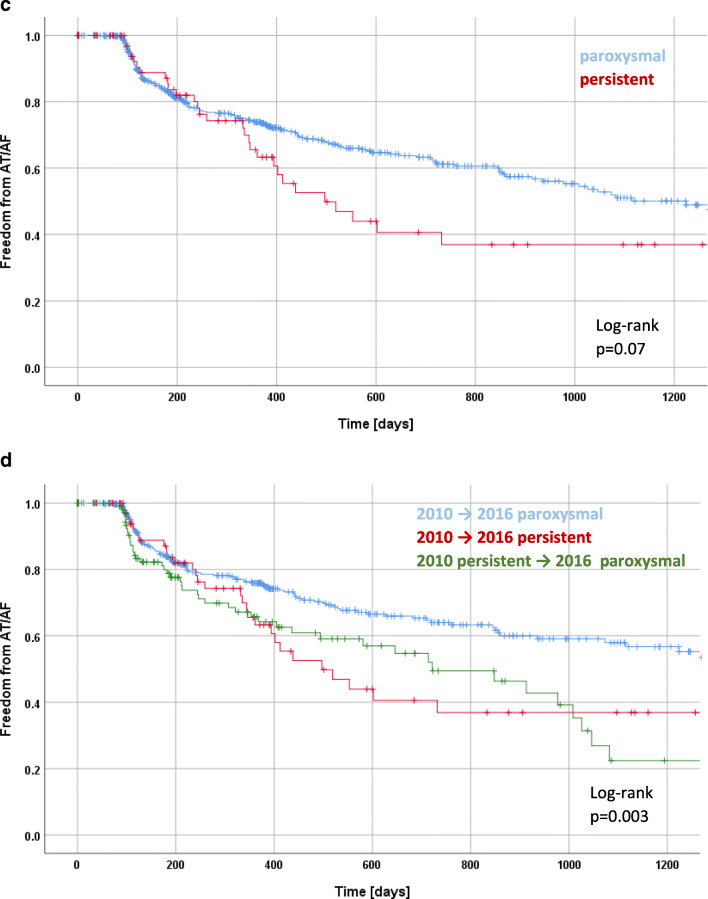
Kaplan-Meier curve of freedom of AF in patients with paroxysmal and persistent AF, categorized according the 2010 ESC guideline definition (**a**), categorized according to the 2012 focused update (**b**), categorized according to the 2016 ESC guideline definition (**c**), showing patients that are paroxysmal and persistent according to the 2010 and 2016 ESC guidelines (**d**), and patients that are reclassified from persistent to paroxysmal according to the 2016 ESC guidelines

According to Kaplan-Meier estimation, recurrence rates of paroxysmal and persistent AF patients according to the 2010 ESC guidelines differ significantly with freedom from AF/AT recurrence in 76% of paroxysmal and 65% of persistent AF patients after 12 months and in 65% of paroxysmal and 46% of persistent AF patients after 24 months (log-rank *p* < 0.001; Fig. 1a). Applying the 2012 ESC focused update, the difference between paroxysmal and persistent AF is not significant with 73% of paroxysmal and 61% of persistent AF patients after 12 months and in 67% of paroxysmal and 49% of persistent AF patients after 24 months (log-rank *p* = 0.13; Fig. 1b). Based on the 2016 ESC guidelines, the difference between paroxysmal and persistent AF is not significant with 74% of paroxysmal and 61% of persistent AF patients after 12 months and in 63% of paroxysmal and 41% of persistent AF patients after 24 months (log-rank *p* = 0.07; Fig. 1c). Kaplan-Meier estimation of freedom from AF/AT recurrence of patients that switch from persistent in the 2010 to paroxysmal in the 2016 guidelines is much more similar to that of patients that maintain persistent AF from the 2010 and 2016 ESC guidelines than those that maintain paroxysmal AF (Fig. 1d).

### Predictors of AF recurrence

Persistent AF has been shown repeatedly to be a predictor of arrhythmia recurrence in PVI studies with mixed, paroxysmal, and persistent cohorts [9, 18]. To evaluate the impact of the re-definition of paroxysmal and persistent AF, we performed a Cox regression analysis to identify potential predictors of AF recurrence in our cohort. Remarkably, applying the 2010 ESC guidelines, only persistent AF is an independent predictor of AF recurrence (Table 4, A). Compared with the 2016 ESC guidelines, persistent AF is no longer a predictor of AF recurrence (Table 4, B).

**Table 4:** Cox regression analysis for AF recurrences according to AF type classification

	Beta coefficient	HR for AF recurrence	*p* value
A. ESC 2010 guidelines			
Female sex	0.039	1.039 (0.743–1.455 95% CI)	0.821
Age (years)	0.008	1.088 (0.991–1.026 95% CI)	0.361
BMI (kg/m^2^)	−0.007	0.993 (0.964–1.022 95% CI)	0.622
Persistent AF 2010 ESC guidelines	0.343	1.409 (1.031–1.961 95% CI)	*0.042*
Hypertension	0.127	1.136 (0.736–1.752 95% CI)	0.565
Dyslipidemia	0.026	1.026 (0.734–1.435 95% CI)	0.880
Diabetes	0.289	1.335 (0.891–2.000 95% CI)	0.162
Coronary artery disease	− 0.001	0.999 (0.713–1.401 95% CI)	0.997
Left atrial diameter (mm)	0.004	1.004 (0.989–1.020 95% CI)	0.582
B. ESC 2016 guidelines			
Female sex	0.015	1.015 (0.726–1.419 95% CI)	0.932
Age (years)	0.009	1.009 (0.992–1.027 95% CI)	0.294
BMI (kg/m^2^)	− 0.007	0.993 (0.965–1.022 95% CI)	0.635
Persistent AF 2016 ESC guidelines	0.225	1.252 (0.809–1.939 95% CI)	0.313
Hypertension	0.137	1.147 (0.744–1.768 95% CI)	0.534
Dyslipidemia	0.021	1.022 (0.730–1.430 95% CI)	0.901
Diabetes	0.295	1.343 (0.895–2.016 95% CI)	0.155
Coronary artery disease	0.020	1.020 (0.727–1.430 95% CI)	0.909
Left atrial diameter (mm)	0.006	1.006 (0.991–1.022 95% CI)	0.420

*AF*, atrial fibrillation; *BMI*, body mass index; *CI*, confident interval; *ESC*, European Society of Cardiology; *HR*, hazard ratio. Significant *p*-value (< 0.05) indicated in italics

## Discussion

In our study, we correlate AF type as defined by current and previous ESC AF guidelines with rhythm outcome of single procedure PVI-only AF ablation. By the redefinition of paroxysmal and persistent AF from the 2010 to the 2016 ESC AF guidelines, almost 20% of all patients switch from the persistent to the paroxysmal group. It is comprehensible that this shift may have an impact on outcome.

Comparing the baseline characteristics of paroxysmal and persistent AF patients according to the 2010 ESC guidelines for the management of AF, its 2012 focused update and the 2016 ESC AF guidelines definition of paroxysmal and persistent AF, persistent AF patients are significantly older, have a lower LVEF, larger LA diameter, and higher CHA_2_DS_2_-VASc score. These findings are consistent in all three classifications and reflect the known risk factors for progression from paroxysmal to persistent AF [15, 16]. Evaluation of patients that switch from persistent to paroxysmal AF after reclassification in comparison with patients that remain paroxysmal or persistent shows that these patients are predominantly male and remarkably have a lower LVEF and larger left atrium than both paroxysmal and persistent AF patients. The reason for this difference is not clear. Nevertheless, one could speculate that patients with reduced LVEF are more prone to developing symptoms immediately after onset of AF and symptoms might be more pronounced than in patients without systolic dysfunction [3, 17]. Thus, it is possible that patients with lower LVEF are more likely to present at the emergency room soon after onset of AF while patients without systolic heart failure tend to present later and more likely in an elective outpatient clinic after more than 7 days than in an immediate emergency setting. In addition, patients with small left atria have a higher probability of spontaneous conversion to sinus rhythm than patients with large left atria. This might explain why patients that have been actively converted to sinus rhythm within 7 days and thus switched classification have larger left atria than patients with spontaneous conversion to sinus rhythm that remain paroxysmal in all guidelines.

The fraction of paroxysmal AF patients with arrhythmia recurrence increases applying 2016 ESC guideline classification in comparison with the 2010 ESC guideline and vice versa, the fraction of persistent patients with arrhythmia recurrence decreases. We show here that the rhythm outcome of patients switching from persistent to paroxysmal is worse than that of patients that are paroxysmal according to both guidelines. In fact, patients that switch classification are more similar to those patients that are persistent according to both guideline definitions than to those patients that are paroxysmal according to both definitions.

Comparison of the Kaplan-Meier estimation of freedom from atrial arrhythmia recurrence shows a significant difference between paroxysmal and persistent patients if the definition of the 2010 ESC guidelines is applied, while the difference between paroxysmal and persistent patients becomes less marked if the definition of the 2012 focused update or the 2016 ESC guidelines is applied rendering this difference non-significant in our study with more than 600 patients.

Surprisingly, it appears that patients that have been classified as persistent AF according to the 2010 ESC guidelines and that are defined as paroxysmal AF in the 2016 guidelines because of active conversion into sinus rhythm early after onset of AF do not have the same favorable outcome after AF ablation as those patients that are defined as paroxysmal in both guidelines. Thus, this challenges the assumption that the majority of these patients would have converted spontaneously. One could speculate that the reason why these patients present early after onset of AF is not only due to variable perception of symptoms but taking into account the significantly lower LVEF, it might be that these patients are more symptomatic due to underlying structural heart disease.

When scheduling paroxysmal AF patients according to the current ESC AF guidelines that would have been persistent AF patients in the 2010 ESC AF guidelines, i.e., patients that have been actively converted to sinus rhythm by antiarrhythmic drugs or by direct current cardioversion within 7 days for AF ablation, the possibility of a less favorable outcome in regard to arrhythmia recurrence should be discussed with patients and referring physicians.

It appears that the discriminatory power regarding rhythm outcome decreases by the redefinition of paroxysmal and persistent AF in the current ESC AF guidelines.

Persistent AF is thought to be caused by substrate such as atrial fibrosis or scar while paroxysmal AF is considered to be mainly driven by triggers with a less pronounced role for atrial substrate. It has been shown that persistent AF is correlated with more substrate than paroxysmal AF both in atrial voltage mapping and in MRI studies [18, 19]. However, probably no clinical definition can provide a perfect consistency with these pathophysiological findings. It would be interesting to analyze the extent of atrial substrate in the patients that switch from persistent to paroxysmal AF. Given our results, one could speculate that these patients might have atrial substrate that is more similar to persistent than to paroxysmal AF patients.

Persistent AF is an independent predictor of AF recurrence in our cohort only if the 2010 ESC guidelines definition is applied. The 2016 ESC guidelines definition does not identify persistent AF as a risk factor for recurrence in our Cox regression model. Hence, the definition of paroxysmal and persistent AF has a predictive implication on studies comparing paroxysmal and persistent AF depending on the definition that has been applied. This should be kept in mind especially when interpolating older results to new studies or more precisely, a clear statement on which definition has been applied should be mandatory for such studies.

### Limitations

Some limitations of this study require consideration.

The study is retrospective and classification of paroxysmal and persistent AF according to the different guidelines was conducted on the basis of medical records. On the other hand, it is a strength of the study that the same cohort is reclassified instead of comparing separate cohorts. The difference in outcome between paroxysmal and persistent AF according to the 2012 focused update and 2016 ESC AF guidelines is non-significant in our study. It cannot be excluded that the difference would still be significant in a larger cohort. Nevertheless, the relevance of a difference that is not significant in a study with more than 600 patients may be questionable.

## Conclusion

Comparing the definition of AF types in the 2010 ESC AF guidelines, the 2012 focused update, and the 2016 ESC AF guidelines, a shift from persistent to paroxysmal AF is recognizable. Patients that switch from persistent to paroxysmal seem to be more similar to those patients that remain persistent throughout the guidelines than to those that remain paroxysmal. As a consequence, it appears that the old definition provided a better separator to predict rhythm outcome. On the other hand, with the current guideline definition and only subtle differences in recurrence rates, it may be challenged whether a strict separation of paroxysmal and persistent AF is justified with regard to the outcome of anticipated AF ablation procedures.

Studies comparing paroxysmal and persistent AF should always clarify according to which guideline type of AF was defined.

## Electronic supplementary material

ESM 1 (DOCX 24 kb)

## References

[1] Camm A, Kirchhof P, Lip G, Schotten U, Savelieva I, Ernst S. ESC Committee for Practice Guidelines. Guidelines for the management of atrial fibrillation: the task force for the management of atrial fibrillation of the European Society of Cardiology (ESC). Europace 2010;12(10):1360–1420. 10.1093/europace/euq350 20876603

[2] Camm A, Lip G, De Caterina R, Savelieva I, Atar D, Hohnloser S (2012). 2012 focused update of the ESC guidelines for the management of atrial fibrillation: an update of the 2010 ESC guidelines for the management of atrial fibrillation–developed with the special contribution of the European Heart Rhythm Association. Europace.

[3] Kirchhof P, Benussi S, Kotecha D, Ahlsson A, Atar D, Casadei B, Castella M, Diener HC, Heidbuchel H, Hendriks J, Hindricks G, Manolis AS, Oldgren J, Popescu BA, Schotten U, van Putte B, Vardas P, Agewall S, Camm J, Baron Esquivias G, Budts W, Carerj S, Casselman F, Coca A, de Caterina R, Deftereos S, Dobrev D, Ferro JM, Filippatos G, Fitzsimons D, Gorenek B, Guenoun M, Hohnloser SH, Kolh P, Lip GY, Manolis A, McMurray J, Ponikowski P, Rosenhek R, Ruschitzka F, Savelieva I, Sharma S, Suwalski P, Tamargo JL, Taylor CJ, van Gelder I, Voors AA, Windecker S, Zamorano JL, Zeppenfeld K (2016). 2016 ESC guidelines for the management of atrial fibrillation developed in collaboration with EACTS. Europace.

[4] De Vos CB, Pisters R, Nieuwlaat R, Prins MH, Tieleman RG, Coelen R-JS (2010). Progression from paroxysmal to persistent atrial fibrillation: clinical correlates and prognosis. J Am Coll Cardiol.

[5] Jahangir A, Lee V, Friedman PA, Trusty JM, Hodge DO, Kopecky SL (2007). Long-term progression and outcomes with aging in patients with lone atrial fibrillation: a 30-year follow-up study. Circulation..

[6] Irfan G, de Asmundis C, Mugnai G, Poelaert J, Verborgh C, Umbrain V (2015). One-year follow-up after second-generation cryoballoon ablation for atrial fibrillation in a large cohort of patients: a single-center experience. Europace.

[7] Hoffmann E, Straube F, Wegscheider K, Kuniss M, Andresen D, Wu L-Q (2019). Outcomes of cryoballoon or radiofrequency ablation in symptomatic paroxysmal or persistent atrial fibrillation. Europace.

[8] Wissner E, Heeger C-H, Grahn H, Reissmann B, Wohlmuth P, Lemes C (2015). One-year clinical success of a “no-bonus” freeze protocol using the second-generation 28 mm cryoballoon for pulmonary vein isolation. Europace.

[9] Cosedis Nielsen J, Johannessen A, Raatikainen P, Hindricks G, Walfridsson H, Kongstad O, Pehrson S, Englund A, Hartikainen J, Mortensen LS, Hansen PS (2012). Radiofrequency ablation as initial therapy in paroxysmal atrial fibrillation. N Engl J Med.

[10] Packer DL, Kowal RC, Wheelan KR, Irwin JM, Champagne J, Guerra PG (2013). Cryoballoon ablation of pulmonary veins for paroxysmal atrial fibrillation: first results of the North American Arctic Front (STOP AF) pivotal trial. J Am Coll Cardiol.

[11] Verma A, Jiang C, Betts TR, Chen J, Deisenhofer I, Mantovan R, Macle L, Morillo CA, Haverkamp W, Weerasooriya R, Albenque JP, Nardi S, Menardi E, Novak P, Sanders P, STAR AF II Investigators (2015). Approaches to catheter ablation for persistent atrial fibrillation. N Engl J Med.

[12] Heeger C-H, Wissner E, Knoell M, Knoop B, Reissmann B, Mathew S (2017). Three-year clinical outcome after 2nd-generation cryoballoon-based pulmonary vein isolation for the treatment of paroxysmal and persistent atrial fibrillation - a 2-center experience. Circ J.

[13] Pott A, Petscher K, Messemer M, Rottbauer W, Dahme T (2016). Increased rate of observed real-time pulmonary vein isolation with third-generation short-tip cryoballoon. J Interv Card Electrophysiol.

[14] Pott A, Kraft C, Stephan T, Petscher K, Rottbauer W, Dahme T (2018). Time-to-isolation guided titration of freeze duration in 3rd generation short-tip cryoballoon pulmonary vein isolation–comparable clinical outcome and shorter procedure duration. Int J Cardiol.

[15] Proietti R, Hadjis A, AlTurki A, Thanassoulis G, Roux J-F, Verma A, Healey JS, Bernier ML, Birnie D, Nattel S, Essebag V (2015). A systematic review on the progression of paroxysmal to persistent atrial fibrillation: shedding new light on the effects of catheter ablation. JACC Clin Electrophysiol.

[16] Ogawa H, An Y, Ikeda S, Aono Y, Doi K, Ishii M, Iguchi M, Masunaga N, Esato M, Tsuji H, Wada H, Hasegawa K, Abe M, Lip GYH, Akao M, Fushimi AF Registry Investigators (2018). Progression from paroxysmal to sustained atrial fibrillation is associated with increased adverse events. Stroke.

[17] Ponikowski P, Voors AA, Anker SD, Bueno H, Cleland JGF, Coats AJS, Falk V, González-Juanatey JR, Harjola VP, Jankowska EA, Jessup M, Linde C, Nihoyannopoulos P, Parissis JT, Pieske B, Riley JP, Rosano GMC, Ruilope LM, Ruschitzka F, Rutten FH, van der Meer P, ESC Scientific Document Group (2016). 2016 ESC guidelines for the diagnosis and treatment of acute and chronic heart failure: the task force for the diagnosis and treatment of acute and chronic heart failure of the European Society of Cardiology (ESC) developed with the special contribution of the Heart Failure Association (HFA) of the ESC. Eur Heart J.

[18] Marrouche NF, Wilber D, Hindricks G, Jais P, Akoum N, Marchlinski F (2014). Association of atrial tissue fibrosis identified by delayed enhancement MRI and atrial fibrillation catheter ablation: the DECAAF study. JAMA.

[19] Rolf S, Kircher S, Arya A, Eitel C, Sommer P, Richter S (2014). Tailored atrial substrate modification based on low-voltage areas in catheter ablation of atrial fibrillation. Circ Arrhythm Electrophysiol.

