# Supplementary material for: Impact of re-definition of paroxysmal and persistent atrial fibrillation in the 2012 and 2016 European Society of Cardiology atrial fibrillation guidelines on outcomes after pulmonary vein isolation
Source: J Interv Card Electrophysiol. 2020 Mar 2;60(1):115–23. doi: 10.1007/s10840-020-00710-4 (PMC7846547; doi:10.1007/s10840-020-00710-4)
Supplement: Supplementary file 1 — (DOCX 24 kb) [file 10840_2020_710_MOESM1_ESM.docx]

**Supplemental Table 1.** Baseline characteristics of patients with paroxysmal and persistent AF classified after the focused update 2012 definition

|  | Total | 2012 ESC focused update | | p |
| --- | --- | --- | --- | --- |
|  | **(n=628)** | **paroxysmal**  **(n=485)** | **persistent**  **(n=143)** |  |
| Female gender | 272 (43) | 211 (44) | 60 (42) | 0.5 |
| Age (years) | 66.2±10.8 | 65.6±11.2 | 68.2±9.0 | **0.005** |
| Hypertension | 457 (79) | 346 (71) | 111 (71) | 0.5 |
| Dyslipidemia | 330 (57.2) | 254 (53) | 76 (53) | 0.7 |
| Diabetes mellitus | 103 (18) | 74 (15) | 29 (20) | 0.2 |
| Coronary artery disease | 206 (36) | 150 (31) | 56 (39) | 0.1 |
| Peripheral vascular disease | 176 (30) | 136 (28) | 40 (28) | 0.7 |
| Oral Anticoagulation | 521 (90) | 390 (95) | 131 (92) | **0.003** |
| Antiarrhythmic drugs |  |  |  |  |
| IC class AAD | 20 (12) | 18 (4) | 2 (1) | 0.5 |
| III class AAD | 11 (6) | 9 (2) | 2 (1) | 1.0 |
| Other drugs |  |  |  |  |
| Beta blockers | 490 (85) | 366 (75) | 124 (87) | **0.006** |
| Claciumantagonists | 133 (23) | 102 (21) | 31 (22) | 1.0 |
| LVEF (%) | 58.7±15.7 | 60.8±15.2 | 52.2±15.7 | **<0.0001** |
| Left atrial size (mm) | 43.2±11.4 | 42.4±11.4 | 45.6±10.6 | **0.005** |
| CHA_2_DS_2_-VASc Score | 2.8±1.6 | 2.7±1.6 | 3.2±1.6 | **0.001** |
| BMI (kg/m^2^) | 28.6±15.7 | 28.5±5.3 | 28.8±5.7 | 0.6 |

Categorical variables are expressed as absolute and percentage (in parentheses). Continuous variables are expressed as mean±SD. AF, atrial fibrillation; AAD, antiarrhythmic drug; BMI, body mass index; LVEF, left ventricular ejection fraction.
